# Palmitic Acid Affects Intestinal Epithelial Barrier Integrity and Permeability In Vitro

**DOI:** 10.3390/antiox9050417

**Published:** 2020-05-13

**Authors:** Manuele Gori, Annamaria Altomare, Silvia Cocca, Eleonora Solida, Mentore Ribolsi, Simone Carotti, Alberto Rainer, Maria Francesconi, Sergio Morini, Michele Cicala, Michele Pier Luca Guarino

**Affiliations:** 1 Gastroenterology Unit, Departmental Faculty of Medicine and Surgery, Università Campus Bio-Medico di Roma, Via Alvaro del Portillo 21, 00128 Rome, Italy; a.altomare@unicampus.it (A.A.); s.cocca@unicampus.it (S.C.); eleonora.solida@gmail.com (E.S.); m.ribolsi@unicampus.it (M.R.); m.cicala@unicampus.it (M.C.); m.guarino@unicampus.it (M.P.L.G.); 2 Microscopic and Ultrastructural Anatomy Unit, Università Campus Bio-Medico di Roma, via Alvaro del Portillo 21, 00128 Rome, Italy; s.carotti@unicampus.it (S.C.); m.francesconi@unicampus.it (M.F.); s.morini@unicampus.it (S.M.); 3 Tissue Engineering Laboratory, Department of Engineering, Università Campus Bio-Medico di Roma, via Alvaro del Portillo 21, 00128 Rome, Italy; a.rainer@unicampus.it

**Keywords:** palmitic acid, Caco-2 monolayer, intestinal permeability, gut barrier integrity, tight junctions, adherens junctions

## Abstract

Palmitic acid (PA), a long-chain saturated fatty acid, might activate innate immune cells. PA plays a role in chronic liver disease, diabetes and Crohn’s disease, all of which are associated with impaired intestinal permeability. We investigated the effect of PA, at physiological postprandial intestinal concentrations, on gut epithelium as compared to lipopolysaccharide (LPS) and ethanol, using an in vitro gut model, the human intestinal epithelial cell line Caco-2 grown on transwell inserts. Cytotoxicity and oxidative stress were evaluated; epithelial barrier integrity was investigated by measuring the paracellular flux of fluorescein, and through RT-qPCR and immunofluorescence of tight junction (TJ) and adherens junction (AJ) mRNAs and proteins, respectively. In PA-exposed Caco-2 monolayers, cytotoxicity and oxidative stress were not detected. A significant increase in fluorescein flux was observed in PA-treated monolayers, after 90 min and up to 360 min, whereas with LPS and ethanol, this was only observed at later time-points. Gene expression and immunofluorescence analysis showed TJ and AJ alterations only in PA-exposed monolayers. In conclusion, PA affected intestinal permeability without inducing cytotoxicity or oxidative stress. This effect seemed to be faster and stronger than those with LPS and ethanol. Thus, we hypothesized that PA, besides having an immunomodulatory effect, might play a role in inflammatory and functional intestinal disorders in which the intestinal permeability is altered.

## 1. Introduction

In the last two decades, several studies have reported the association between disruption of intestinal permeability and different clinical disorders, such as inflammatory bowel disease (IBD), celiac disease, irritable bowel syndrome (IBS), diabetes, and obesity [1,2]. A growing number of molecules and mechanisms involved in intestinal barrier function have been studied and identified in animals, patient specimens, and in vitro models of the gut epithelium [3,4,5,6,7]. Nevertheless, the exact mechanisms underlying the pathophysiology of gastrointestinal (GI) disorders and impaired intestinal permeability are not clearly established.

It has been widely shown, both in humans and in animal models, that intestinal barrier function is highly sensitive to stress, dietary, and microbiota changes [8,9,10]. Changes in the microbiota population have been reported in GI diseases such as IBS and IBD [1,2], in which the integrity of the intestinal barrier is known to be impaired [9,11,12,13], although the mechanisms underlying microbiota modifications and altered intestinal permeability are still not precisely understood. Increased intestinal permeability could lead to a translocation of the luminal content (such as bacteria and pathogenic molecules) to the bloodstream, as demonstrated in GI chronic diseases, acute intestinal failure, and gram-negative sepsis [14,15,16].

Furthermore, it was reported that the bacterial lipopolysaccharide (LPS), an endotoxin originating from the cell wall of Gram-negative bacteria [17,18], which is known to be a potential mediator of multisystem organ failure in the course of endotoxemia, at high pharmacological concentrations, causes rapid cell death in various cell types [19,20]. Additionally, at lower concentrations, LPS causes a selective increase in the intestinal tight junction (TJ) permeability in vitro as well as in vivo [21]. In previous studies, using an ex vivo experimental model, we assessed how acute exposure of colonic mucosa to pathogenic LPS impairs the contractility of human colonic smooth muscle cells (HCSMCs), owing to both LPS mucosal translocation and production of free radicals, thus supporting the hypothesis that augmented permeability might contribute to the onset of GI disease [5,6].

In recent years, a growing interest in understanding the role of lifestyle-related factors in disrupting the intestinal barrier functions has emerged, and the effect of several dietary products are now the object of several studies [1,22,23].

Over the past decade, we have learned about the adverse effects of ethanol intake on the functional and structural integrity of the intestinal mucosa, with loss of the intestinal barrier function [24,25,26].

Among the dietary products, palmitic acid (PA) has also drawn much attention in the scientific community, due to its well-known lipotoxic effects on different organs in humans [27,28,29]. PA is the most common long-chain saturated (16:0) fatty acid found in animals, plants, and microorganisms [30]. At the intestinal level, PA is known to modulate the immune system by inducing monocyte activation and stimulating pro-inflammatory responses in human immune cells [31,32]. Furthermore, PA impairs intestinal insulin sensitivity [33], and is associated with an increased incidence of type 2 diabetes [34,35]. At the hepatic level, gut-microbiota-derived PA in a mouse model of steatohepatitis-inducing high-fat diet, was responsible for activating liver macrophages and promoting the TNF-α expression, thus, contributing to the pathogenesis of non-alcoholic steatohepatitis [36]. Until now, PA has been studied in relation to Caco-2 cells, only with regards to its metabolism and uptake across the Caco-2 monolayer [37,38,39], but till date, not much is known about the effect of PA on the intestinal barrier function. Hence, in the present work we aimed to investigate this aspect by comparing the effect of PA at physiological postprandial intestinal concentrations [37,40], on the intestinal barrier integrity, with agents known from the literature, in particular the aforementioned LPS and ethanol.

## 2. Materials and Methods

### 2.1. Cell Culture

Human epithelial colorectal adenocarcinoma (Caco-2) cells purchased from the American Type Culture Collection (ATCC, VA, USA), were maintained under standard cell culture conditions, in a humidified 37 °C incubator, with 5% CO_2_, in Dulbecco’s modified eagle medium (DMEM high glucose, Euroclone, Italy) containing 10% (vol/vol) fetal bovine serum (FBS, Lonza, Switzerland), 2 mM L-glutamine, 100 U/mL penicillin, and 100 μg/mL streptomycin (both from Euroclone). Cells seeded at a density of 6.0 × 10^4^ per insert were grown on transwell chambers (12 mm with 0.4 μm pore polyester membrane inserts; Corning, Sigma-Aldrich, Milan, Italy) placed in a 12-well plate. The cells were regularly monitored using a fully motorized epifluorescence inverted microscope (Eclipse Ti-E, Nikon, Japan), by measuring the epithelial resistance. Experiments were performed 21 days after seeding when the cells reached confluence and differentiation. Fresh media was changed every other day in the apical and basolateral compartments of the well, until the day of experimentation.

### 2.2. Treatments with LPS, PA, and Ethanol

Cells were challenged apically with different chemicals for 24 h (bacterial lipopolysaccharide from a pathogenic strain of Escherichia Coli 0111:B4 and PA) and 1 h (ethanol) at 37 °C. LPS (10 μg/mL, *w/v*, Sigma-Aldrich, Milan, Italy) [41] was dissolved in plain DMEM. PA (1 mM, *w/v*, Sigma-Aldrich, Milan, Italy) was dissolved in methanol (vehicle) as 50 mM stock solution, and then diluted in a free fatty acid (FFA) medium that was DMEM supplemented with 2 mM L-glutamine (Euroclone), 1% bovine serum albumin (BSA) Cohn fraction V (Sigma-Aldrich, Milan, Italy), and 10% charcoal-stripped FBS (Hyclone, GE Healthcare, Chicago, IL, USA), as previously reported [42]. The concentration of 1 mM for 24 h—which is a common time-point used in LPS and PA experiments [17,19,38,39,40,41,43]—was chosen as a physiological postprandial intestinal concentration, as per the literature [37,40] and because it was within the concentration range of FFAs in human plasma (i.e., 0.2–2 mM) [44] used in our and other previous works on developing in vitro models of hepatic steatosis [45,46,47,48]. For PA treatments, internal controls were represented by cells cultured in the FFA medium with the same volume of vehicle (methanol). When the ethanol effect was investigated, we exposed only the apical side of the cells to a 10% ethanol solution (*v/v*) for 1 h in complete DMEM [49,50], as ethanol is primarily present in the gut lumen after ingestion. Likewise, LPS and PA were dispensed only in the apical side as, once in the lumen, they are both absorbed by the microvilli covering the apical surface of the mucosa [41,51]. After treatments, the cells were rinsed in phosphate buffered saline w/o Ca^2+^ and Mg^2+^ (PBS, Euroclone, Milan, Italy), and used for different assays. Undernatants were collected for further analysis, as described below.

### 2.3. Analysis of Cell Viability/Cytotoxicity

After challenges with different chemicals, Caco-2 monolayers were rinsed in PBS and incubated with the ReadyProbes^®^ Cell Viability Imaging Kit (Blue/Green, Thermo Fisher Scientific, Waltham, MA, USA), for 30 min (min) at 37 °C in complete FluoroBrite DMEM (Gibco, Thermo Fisher Scientific, Waltham, MA, USA). In brief, the NucBlue^®^ Live reagent stained the nuclei of all cells, whereas the NucGreen^®^ Dead reagent selectively detected only the nuclei of dead cells, with compromised plasma membranes. Micrographs were acquired and analyzed using the Eclipse Ti-E (Nikon, Tokyo, Japan), equipped with a high-sensitivity camera (Neo 5.5, Andor, Belfast, Ireland) and automated acquisition/analysis software (NIS Elements AR, Nikon, Tokyo, Japan). Cell viability was determined by counting the total vs. dead cells in at least three microscopic random fields/well. Results were plotted as a percentage of live cells in the treated vs. control cultures referred to as 100% in the graphs. Cell viability/cytotoxicity was further assessed using a Vybrant Cytotoxicity Assay Kit (Thermo Fisher Scientific, Waltham, MA, USA), as described previously [52]. In brief, 50 μL of the medium was collected after each treatment and transferred into a 96-well microplate; 50 μL of the reaction mixture was added, and the microplate was incubated at 37 °C for 15 min. The microplate was read on a Tecan Infinite M200-Pro fluorescence microplate reader (Tecan, Männedorf, Switzerland), with ex/em 535/595 nm. Cytotoxicity was calculated as the fluorescence intensity ratio between the experimental group and the fully lysed control, both corrected for background using the no-cell negative control. In all experiments, 2% Triton X-100 was used as a positive control for 2 h [7,50].

### 2.4. Analysis of Oxidative Stress

Oxidative stress was measured by assessing the intracellular reactive oxygen species (ROS) levels generated after exposure to LPS, ethanol, and PA, through the green-fluorescent ROS detection reagent 6-carboxy-2’,7’-dichlorodihydrofluorescein diacetate, di-(acetoxymethyl ester) (carboxy-H_2_ DCFDA, Thermo Fisher Scientific, Waltham, MA, USA), according to the method described in [42], with slight modifications. In brief, the cells were rinsed in PBS and loaded with 10 μM of the cell-permeant probe carboxy-H_2_ DCFDA for 30 min at 37 °C, in complete FluoroBrite DMEM (Gibco, Thermo Fisher Scientific, Waltham, MA, USA), to exclude hydrogen peroxide generation in a medium containing phenol red, before fluorescence analysis. Incubation with 400 μM H_2_O_2_ (Sigma-Aldrich, Milan, Italy) for 3 h was used as positive control for ROS [53,54]. After incubation with carboxy-H_2_ DCFDA, the cells were rinsed in PBS and left in fresh FluoroBrite DMEM for a recovery time of 1 h. Images were acquired under an epifluorescence inverted microscope (Nikon, Tokyo, Japan). The ROIs occupied by cells were identified from phase contrast micrographs and used for fluorescence analysis. Mean fluorescence intensity (MFI) of the positive cells (FITC filter set) was quantified through the NIS Elements AR software (Nikon). All treated cells were normalized to their own internal controls, after background subtraction. Representative graphs were intensity surface plots of each treatment.

### 2.5. Cell Permeability Assay

Epithelial barrier function was assessed by measuring unidirectional paracellular flux of fluorescein isothiocyanate-dextran 4000 (FD-4, Sigma-Aldrich, Milan, Italy) from the apical to basolateral compartments. This assay was conducted by slightly modifying the protocol from [50]. In brief, prior to transport studies, the culture medium on both sides of the Caco-2 cell monolayer was removed by aspiration and replaced with prewarmed Krebs-Henseleit Buffer (KHB, pH 7.4) for 1 h at 37 °C. The cells were then washed in PBS, and FD-4 was applied to the apical side of the Caco-2 monolayer, at 1 mg/mL final concentration in prewarmed KHB [4], and its paracellular permeability was calculated from the apical to basolateral direction by collecting the undernatants, as previously described [55]. The concentration of FD-4 in the solution was measured every 30 min over 6 h, by removing an aliquot from the receiver compartment (undernatants) and replacing it with an equal volume of fresh KHB [4]. To evaluate the reversibility of the PA effect, washout experiments were performed, as described elsewhere [50,56]. In brief, Caco-2 cell monolayers, previously treated with PA at 1 mM for 24 h, were thoroughly washed with PBS and then exposed to PA-free Caco-2 DMEM for an additional 24 h. At the end of the recovery period, the passage of FD-4 across the Caco-2 cell monolayer was evaluated, as described above. Fluorescence readings were carried out at ex/em 490/520 nm by using a multiplate reader (Tecan). Fluorescence values were converted in concentrations of fluorescein (pmol), using a standard curve. Each experiment was performed at least in triplicates with independent controls among the three conditions.

### 2.6. Gene Expression Analysis of Cell-to-Cell Adhesion Proteins

Expression of TJ and adherens junction (AJ) mRNAs was evaluated through quantitative reverse transcription polymerase chain reaction (RT-qPCR), using the comparative cycle threshold (Ct) method of relative quantification (ΔΔCt). Isolation of mRNA was performed using acid guanidinium thiocyanate–phenol–chloroform extraction (TRIzol, Thermo Fisher Scientific). Extracted mRNA was quantified spectrophotometrically (Nanodrop, Thermo Fisher Scientific, Waltham, MA, USA) and reverse-transcribed using the High Capacity cDNA Reverse Transcription kit (Thermo Fisher Scientific, Waltham, MA, USA), according to the manufacturer’s instructions. Amplification was performed on 20 ng of cDNA in a total reaction volume of 10 μL on an ABI 7900HT Fast Real-Time PCR System (Thermo Fisher Scientific, Waltham, MA, USA) using the TaqMan universal Master Mix II (Thermo Fisher Scientific, Waltham, MA, USA) and TaqMan Gene Expression Assay primers (Thermo Fisher Scientific, Waltham, MA, USA) for TJP1 (Hs01551861_m1); CDH1 (Hs01023894_m1); and CTNNB1 (Hs00355049_m1). After verifying their stable expression through the geNorm software [57], 18S rRNA (18S, Hs99999901_s1) was used as the endogenous control.

### 2.7. Immunohistochemistry of Cell-to-Cell Adhesion Proteins

To assess the epithelial barrier integrity, immunofluorescence stainings against cell-to-cell adhesion proteins (i.e., TJ and AJ proteins) were performed using the following primary antibodies—anti-Zonula Occludens-1 (ZO-1, also known as TJP1, IgG rabbit polyclonal, 21773-1-AP, Proteintech Group Inc., Rosemont, IL, USA) diluted 1:50; anti-E-cadherin (CDH1, IgG1 mouse monoclonal, sc-8426, Santa Cruz Biotechnology Inc., Dallas, TX, USA) diluted 1:50; and anti-β-catenin (CTNNB1, IgG1 mouse monoclonal, sc-7963, Santa Cruz Biotechnology Inc., Dallas, TX, USA) diluted 1:50. All antibody dilutions were done in BlockAid blocking solution (Thermo Fisher Scientific, Waltham, MA, USA). Indirect immunostainings were done as follows—Caco-2 cell monolayers were washed in PBS and fixed in 4% paraformaldehyde in PBS, for 15 min at room temperature (RT). To block prolonged fixation, the samples were washed in 1 mM glycine, and subsequently permeabilized with ice cold methanol for 5 min. After washing in PBS, the monolayers were blocked in BlockAid (Thermo Fisher Scientific, Waltham, MA, USA), for 45 min at RT. Incubation with primary antibodies was done overnight at 4 °C. After careful washing with PBS, goat anti-rabbit IgG (H + L) Alexa Fluor-488 and goat anti-mouse IgG (H+L) Alexa Fluor-488 (Thermo Fisher Scientific, Waltham, MA, USA) secondary antibodies were incubated for 1 h at RT, in the dark in BlockAid (Thermo Fischer Scientific, Waltham, MA, USA). Nuclei were counterstained with DAPI (1:10000 for 10 min, 62248, Thermo Fischer Scientific, Waltham, MA, USA). The samples were rinsed three times in PBS before mounting on microscope glass slides (Menzel Gläser, Thermo Fisher Scientific, Waltham, MA, USA), and were covered with ProLong Diamond Antifade Mountant (P36961, Thermo Fisher Scientific, Waltham, MA, USA). Images were captured using an epifluorescence inverted microscope (Nikon, Tokyo, Japan) and analyzed through the NIS Elements AR software (Nikon, Tokyo, Japan) for evaluating the continuity of distribution of each protein on the cell surface, and quantifying their corresponding fluorescence intensity by counting three microscopic random fields per well. Each experiment was performed in triplicate.

### 2.8. Statistical Analysis

Data are presented as mean ± standard error of the mean (SEM), of at least three independent experiments. Data were analyzed using Origin v. 9 (OriginLab Corp., Northampton, MA, USA) and GraphPad Prism v. 6, (La Jolla, San Diego, CA, USA) software tools. Normally distributed data were analyzed for significance by unpaired *t*-test and two-way ANOVA, followed by post-hoc test (Bonferroni’s), when appropriate. Significance was at the 0.05 level.

## 3. Results

### 3.1. Effect of the Different Treatments with LPS, PA, and Ethanol on the Caco-2 Cell Monolayer Viability/Cytotoxicity

**Figure 1:**
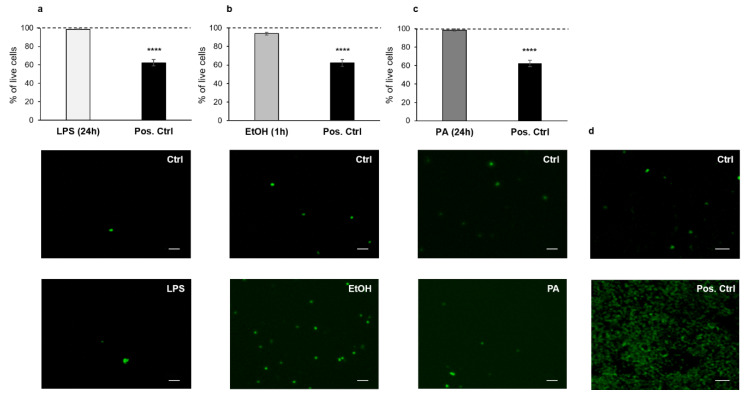
Analysis of cell viability/cytotoxicity following the treatments of Caco-2 monolayers with lipopolysaccharide (LPS), ethanol, and palmitic acid (PA). (**a**) LPS (10 μg/mL) for 24 h. (**b**) Ethanol (10%) for 1 h. (**c**) PA (1 mM) for 24 h. The histograms show the percentage of living cells for the control (dashed lines, referred to as 100%), treated (grey bars), and positive control cells (black bars, by using 2% Triton X-100 for 2 h) of human colonic mucosa. Representative micrographs of the different treatments show the nuclei of dead cells (green), through the Blue/Green Cell Viability Imaging Kit, for both the treated (lower panels in **a**–**d**) and the control cells (middle panels in **a**–**d**). Each treatment was compared to its own internal control. Data are reported as mean ± SEM; *n* = 3 independent experiments. Statistical analysis was performed using the unpaired *t*-test. **** *p* < 0.0001. Scale bars: 50 μm. Ctrl (Control); LPS (Lipopolysaccharide); EtOH (Ethanol); PA (Palmitic Acid); and Pos. Ctrl (Positive Control).

Caco-2 cell viability and cytotoxicity of the treatments were first evaluated using the ReadyProbes^®^ Cell Viability Imaging Kit, under an epifluorescence inverted microscope. For all three chemicals used, there was no significant difference in cell viability (%) between the treated cell monolayers (representative fluorescence micrographs in Figure 1a–c, lower panels) and their respective internal controls (representative fluorescence micrographs in Figure 1a–c, middle panels). The observed values were around 99% for LPS (98.73 ± 0.48%, histogram in Figure 1a) and PA (98.47 ± 1.22%, histogram in Figure 1c), and 93.88 ± 1.35% for ethanol (histogram in Figure 1b), unlike the positive control (Pos. Ctrl, 2% Triton X-100 in Figure 1a–c, black bars and representative fluorescence micrograph in Figure 1d, lower panel), which showed a statistically significant difference compared to the control (62.29 ± 3.51%, *p* < 0.0001, representative fluorescence micrograph in Figure 1d, middle panel). To further assess the cytotoxicity induced by the treatments, we also analyzed Caco-2 monolayers through the Vybrant Cytotoxicity Assay, which confirmed the cell viability results, with a cell survival higher than 99% in all treatments (i.e., 99.22 ± 0.18% for LPS; 99.09 ± 0.32% for PA; 99.66 ± 0.09% for ethanol), except for the positive control (2% Triton X-100) with a 65.89 ± 1.71% cell survival, *p* < 0.0001, vs. control (data not shown).

### 3.2. Analysis of ROS Production Following Treatments

**Figure 2:**
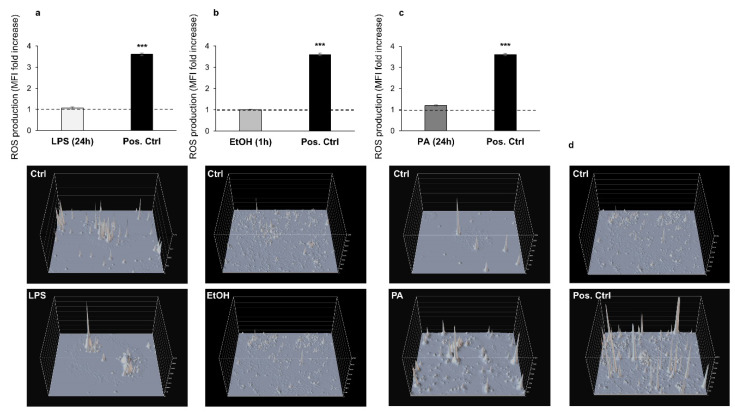
Reactive oxygen species (ROS) production for the analysis of oxidative stress following the treatments of Caco-2 monolayers with LPS, ethanol, and PA. The histograms show the fold increase of MFI expressed as the ratio between the treated (grey bars) and control (dashed lines, referred to as 1) cells, for LPS (**a**), ethanol (**b**), and PA (**c**); positive control cells (black bars in **a**–**c**) were exposed to 400 μM H_2_O_2_ for 3 h. Representative intensity surface plots of the control and treated cells for LPS (in **a**, middle and lower panels, respectively), ethanol (in **b**, middle and lower panels, respectively), PA (in **c**, middle and lower panels, respectively), and positive control (in **d**, middle and lower panels, respectively). Data are reported as mean ± SEM; *n* = 3 independent experiments. Statistical analysis was performed using unpaired *t*-test. *** *p* < 0.001. Ctrl (Control); LPS (Lipopolysaccharide); EtOH (Ethanol); PA (Palmitic Acid); and Pos. Ctrl (Positive Control).

With the aim of investigating the oxidative stress caused by exogenous exposure to the three chemicals, we evaluated intracellular ROS levels in LPS-, ethanol-, and PA-treated Caco2 monolayers, via the carboxy-H_2_ DCFDA fluorescent probe, according to previous works [58,59]. Caco-2 monolayers exposed to H_2_O_2_ [53,54] were considered as positive controls (Figure 2d). ROS levels generated in the Caco-2 monolayers, normalized to their internal controls, were negligible and comparable in all treatments—LPS (1.07 ± 0.025 vs. control), ethanol (1.01 ± 0.010 vs. control), and PA (1.2 ± 0.034 vs. control) (Figure 2).

### 3.3. FD-4 Permeability Analysis

**Figure 3:**
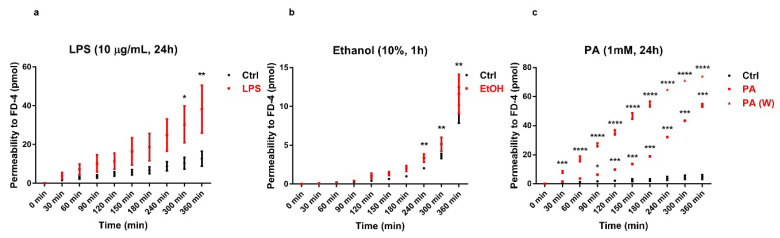
Barrier function of the Caco-2 cell monolayers analyzed through the FD-4 permeability assay. (**a**) LPS treatment (10 μg/mL) for 24 h. (**b**) Ethanol treatment (10%) for 1 h. (**c**) PA treatment (1 mM) for 24 h and washout (W), with a 24 h recovery after PA treatment (1 mM) for 24 h. The amount of FD-4 (expressed in pmol) accumulated in the receiver compartment, was plotted as a function of time (expressed in min)—in red for each treatment (square dots and triangular dots) and in black for the internal control (round dots)—with independent controls among the three conditions. Data are reported as mean ± SEM; n = 4 independent experiments. Statistical analysis was performed using a two-way ANOVA, followed by Bonferroni’s post-hoc correction test. * *p* < 0.05, ** *p* < 0.01, *** *p* < 0.001, **** *p* < 0.0001. Ctrl (Control); LPS (Lipopolysaccharide); EtOH (Ethanol); PA (Palmitic Acid); and W (Washout).

Previous studies [49,50,60,61], using the Caco-2 cell monolayer model, have demonstrated an inverse relationship between intestinal epithelial resistance and paracellular permeability, after exposure to various insults. To evaluate the alteration of the Caco-2 cell monolayer integrity, we measured the paracellular penetration amount of FD-4 across Caco-2 monolayers. FD-4 is a large molecule with a molecular weight of 4 kDa. The cumulative increase of FD-4 fluorescence in the receiver compartment was measured using a multiplate reader (Tecan), calculated and plotted as a function of time, every 30 min over 6 h (from 30 min up to 360 min in Figure 3), similar to [4]. In all treatments, FD-4 permeability across the epithelial barrier increased when compared to their own internal controls (Figure 3a–c). In detail, the elevation of permeability induced by LPS at 10 μg/mL for 24 h (Figure 3a), became statistically significant only after 300 min (*p* < 0.05) and at 360 min (*p* < 0.01), and was in agreement with the literature [4,17,19,41]. The treatment with 10% ethanol for 1 h also induced a general trend of increase in FD-4 permeability (Figure 3b), which became statistically significant from 240 min (*p* < 0.01) onwards, compared to its internal control. Remarkably, upon treating cells with PA at 1 mM for 24 h, there was an increase in FD-4 permeability (Figure 3c), which was statistically significant already after 90 min (*p* < 0.05) and up to 360 min (*p* < 0.001), compared to the internal control. To evaluate the persistence of the PA-induced effect on the permeability to FD-4, we performed a washout experiment and observed that a 24 h recovery period did not restore the original epithelial barrier integrity (Figure 3c). In fact, permeability to FD-4 increased after PA exposure in a statistically highly significant manner, after 30 min (*p* < 0.001), as compared to its internal control and even more significantly over time (*p* < 0.0001, from 60 min to 360 min). Therefore, the effect of PA on intestinal permeability was not reversible.

### 3.4. RT-qPCR Analysis of TJ and AJ Complexes

**Figure 4:**
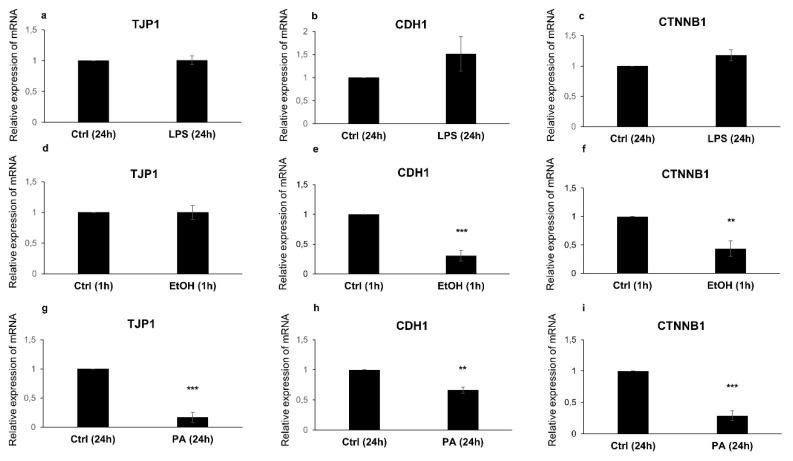
Gene expression analysis of the intestinal barrier complexes. Gene expression of tight junction protein 1 (TJP1), E-cadherin (CDH1), and β-catenin (CTNNB1) in the Caco-2 monolayers treated with LPS for 24h (**a**–**c**), with ethanol for 1 h (**d**–**f**), and with PA for 24 h (**g**–**i**), compared to the internal controls. Data are presented as relative expression of mRNAs vs. controls and reported as mean ± SEM; *n* = 3 independent experiments. Statistical analysis was performed using the unpaired *t*-test. ** *p* < 0.01 and *** *p* < 0.001. Ctrl (Control); LPS (Lipopolysaccharide); EtOH (Ethanol); and PA (Palmitic Acid).

Next, the gene expression patterns of the tight junction protein TJP1, and the adherens junction proteins E-cadherin and β-catenin were evaluated after the different treatments. In the LPS-treated cells, the expression of the mRNAs of TJP1, E-cadherin, and β-catenin, was not significantly affected as compared to the control cells (Figure 4a–c, respectively).

In ethanol-treated cells (Figure 4), only the mRNAs expression of the AJ genes E-cadherin (CDH1 in Figure 4e) and β-catenin (CTNNB1 in Figure 4f) was significantly downregulated, compared to the control cells (*p* = 0.00069 and *p* = 0.0073, respectively). However, the mRNA expression of TJP1 was not downregulated (Figure 4d). Remarkably, in the Caco-2 cells treated with PA, both TJ and AJ genes were modulated compared to the control cells (Figure 4). The TJP1 mRNA expression (Figure 4g), together with E-cadherin (CDH1 in Figure 4h) and β-catenin (CTNNB1 in Figure 4i) mRNAs, were significantly lower than the control cells (*p* = 0.00030, *p* = 0.00145, and *p* = 0.00039, respectively).

### 3.5. Immunohistochemical Analysis of TJ and AJ Proteins

**Figure 5:**
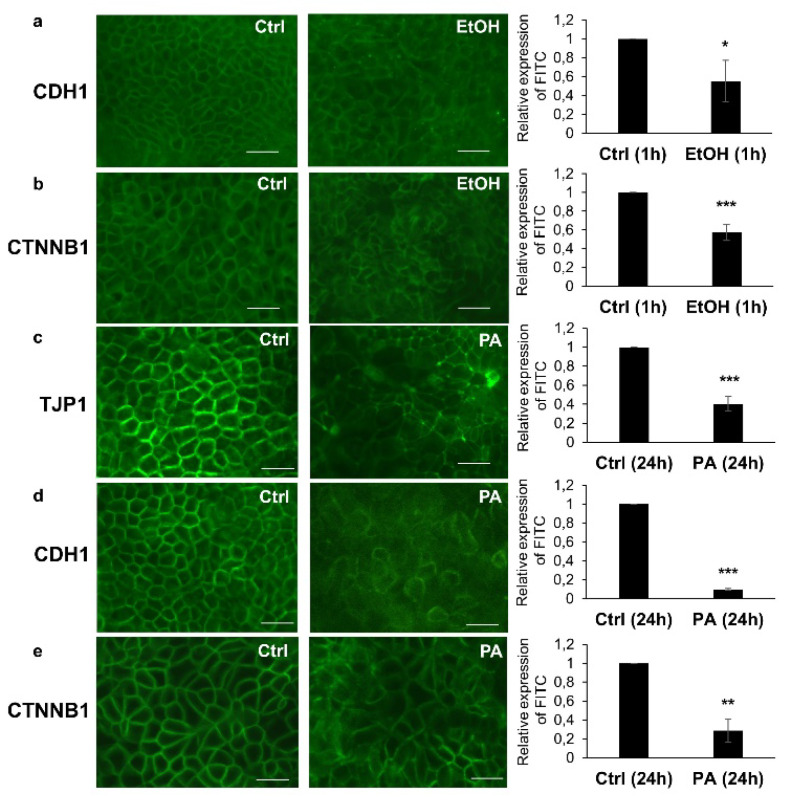
Immunohistochemical analysis of the intestinal barrier proteins. Differential protein expression of E-cadherin (CDH1) and β-catenin (CTNNB1) between the control (**a**,**b**, far left panels) and ethanol-treated (**a**,**b**, middle panels) Caco2 cells, with a discontinuous expression on the cell surface, as shown in the representative micrographs; in (**a**, far right panel) and (**b**, far right panel) the histograms show the relative expression of the fluorescence intensity (FITC) on the cell surface of the Caco-2 monolayers treated with ethanol vs. the controls, through perimeter analysis. Differential expression of tight junction protein 1 (TJP1), CDH1, and CTNNB1 between the control (**c**,**d**,**e**, far left panels, respectively) and the PA-treated (**c**,**d**,**e**, middle panels, respectively) Caco2 cells; in (**c**), (**d**), and (**e**) the far right histograms show the relative expression of the fluorescence intensity (FITC) on the cell surface of Caco-2 monolayers treated with PA vs. the controls, as for the far right histograms of (**a**) and (**b**). The immunostainings against CDH1 in the control (**d**, far left panel) and PA-treated (**d**, middle panel) Caco-2 cells also highlight the different morphology and size of the PA-treated vs. control cells. Data are reported as mean ± SEM; *n* = 3 independent experiments. Statistical analysis was performed using unpaired *t*-test. * *p* < 0.05, ** *p* < 0.01, *** *p* < 0.001. Scale bars: 25 μm. Ctrl (Control); EtOH (Ethanol); and PA (Palmitic Acid).

Finally, we analyzed the protein expression of the two multiprotein junctional complexes through immunohistochemistry. Thus, the pericellular distribution and arrangement of TJP1, CDH1, and CTNNB1 were assessed at the Caco-2 cellular membranes using immunofluorescence microscopy (Figure 5). LPS-treated cells, in agreement with the gene expression data, did not show any differential expression or discontinuous distribution of TJP1, CDH1, and CTNNB1, compared to the control cells (Figure S1a–C, respectively). Unlike the LPS, cells treated with ethanol showed a discontinuous pericellular expression of both CDH1 (Figure 5a, middle panel) and CTNNB1 (Figure 5b, middle panel), but not TJP1 (Figure S1d). The ethanol-treated cells also showed a weaker fluorescence intensity compared to a more marked and homogeneous distribution of the two proteins on the surface of the control cells (Figure 5a,b, far left panels, respectively), whose difference was statistically significant (histograms in Figure 5a,b, respectively) and in line with the corresponding gene expression data (Figure 4e,f, respectively).

Interestingly, only PA was able to induce a discontinuous pericellular distribution of TJP1, CDH1, and CTNNB1 (as shown in Figure 5c–e, middle panels, respectively) compared to the controls (Figure 5c–e, far left panels, respectively), showing statistically significant differences between the two treatments (histograms in Figure 5c–e, respectively). The RT-qPCR analysis in the PA-treated cells vs. the controls showed analogous results for the gene expression of the three proteins (Figure 4g–i). Moreover, the immunostaining against CDH1 showed a different cell morphology and an enlarged size in the PA-treated cells (Figure 5d, middle panel), which appeared to be more disconnected with each other, as compared to the tighter and more compact monolayer of the control cells (Figure 5d, far left panel).

## 4. Discussion

In the present work, the direct effect of PA at a physiological postprandial intestinal concentration on the integrity of gut epithelium was tested, as compared to bacterial lipopolysaccharide and ethanol, using a Caco-2 cell model. The human intestinal epithelial cell line Caco-2, a colonic adenocarcinoma cell line, after post-confluent differentiation and polarization exhibits similar functional and structural characteristics to normal human intestinal epithelium (including formation of TJ) and has been widely used for investigating the intestinal responses to drugs and toxins [8,62].

Our evidence showed that the concentrations of the three substances used did not induce any toxic insult to the Caco-2 cell monolayers, in agreement with previous works, at least as far as LPS and ethanol [41,49,61,63]. Moreover, the concentrations of the three substances used in this study were not elevated enough to cause an oxidative stress damage to the intestinal epithelium. Conversely, FD-4 permeability across the epithelial barrier increased, when compared to their own internal controls in all treatments, with a remarkable augmentation upon treating the cells with PA. Interestingly, such effect seems to be faster and even more significant than those detected following LPS and ethanol exposure, and notably did not show any sign of reversibility, even after a 24 h recovery period following PA insult. Related to these findings, it has been previously shown that ethanol in high doses (≥ 40%) induces cytotoxic effects on mucosa cells, by causing cell death of the gastrointestinal mucosal surface [64]. Whereas, at low doses (≤ 10%), ethanol is not cytotoxic and causes a functional and structural opening of the Caco-2 intestinal epithelial TJ barrier [49]. Indeed, it has been demonstrated that a range of ethanol concentrations (e.g., 2.5–15%) reduces the transepithelial electrical resistance (TEER) and increases the paracellular permeability of Caco-2 monolayers [65]. Similarly, although pure LPS does not pass across the healthy intestinal barrier in vivo as well as in vitro [66,67], several studies have shown that, at physiological and clinical concentrations, biologically active LPS causes a significant increase in the paracellular permeability of gut epithelium in a Toll-like receptor 4 (TLR4)-dependent manner, by downregulating the expression of TJ-related genes and proteins, but without intestinal epithelial cell death [4,21,41,63].

The role of TJ and AJ protein complexes in the increased Caco-2 monolayer permeability observed above was also evaluated, as it is known that these proteins play a pivotal role in epithelium formation and maintenance of tissue integrity, as well as in the regulation of TJ and AJ permeability [68,69]. Defective intestinal epithelial TJ barrier, characterized by an increase in intestinal permeability, was shown to be an important pathogenic factor that contributes to the development of IBD and necrotizing enterocolitis (NEC) [70,71]. Immediately below the TJs are the cadherin-dependent AJs, necessary for the correct assembly of the TJ complexes [72,73,74,75], which regulate the cell-to-cell adhesions that are pivotal to the formation of the intestinal epithelial barrier [76,77,78]. In particular, one of the main components of such protein complexes, the E-cadherins that mediate the cell–cell adhesions of AJs, seem to be required for the epithelial barrier function, and the signals that are transmitted through the AJs can also regulate TJs [72,79,80]. Hence, gene expression analysis of the TJ protein TJP1, and the AJ proteins E-cadherin and β-catenin was done after the different treatments. Interestingly, in the Caco-2 cells treated with PA, both the TJ and AJ genes were modulated, compared to the control cells with a significantly lower expression of TJP1, together with E-cadherin and β-catenin. The same alterations were not observed in the LPS-treated cells in which the expression of the mRNAs of TJP1, E-cadherin, and β-catenin was not significantly affected, compared to the control cells; while the ethanol treatment only downregulated the expression of the AJ genes, E-cadherin and β-catenin.

Finally, immunohistochemical analysis showed that only PA was able to induce a discontinuous pericellular distribution of TJP1, CDH1, and CTNNB1, compared to the controls. Moreover, the immunostaining against CDH1 showed a diverse cell morphology and an enlarged size in the PA-treated cells, which appeared to be slightly looser with each other, as compared to the tighter and more compact monolayer of the control cells. As in the case of LPS, PA is known to activate proinflammatory pathways in various tissues, including the gut, via transmembrane receptors such as TLR4 [20,31,32,35], as well as through the alteration of gut microbiota [81], leading to increased systemic levels of inflammatory cytokines [31,32,35,82]. In such a scenario, TLR4 seems to be the connection hub among the consumption of dietary fats, metabolic inflammation, and insulin resistance [83]. Thus, we can speculate that the increased gut permeability observed in our study, which was due to the PA-dependent physical alteration of the intestinal barrier, could pave the way to the above-mentioned inflammatory pathways, thereby favoring the downstream release of cytokines and other proinflammatory factors reported in the literature [31,32,35,82,83]. This hypothesized mechanism deserves a future investigation in order to be confirmed.

Based on the data obtained from cell viability, oxidative stress, and permeability assays, we can infer that, the herein tested concentration (1 mM) of PA affects the intestinal permeability without inducing cytotoxicity or oxidative stress. PA insult on the integrity of gut epithelium seems likely to cause a functional change in the TJ and AJ barrier, through a putative effect on the disassembly of the TJ and AJ proteins (i.e., paracellular gap opening), as reported previously [82], rather than through a permanent cell damage or cell death effect, as also confirmed by the gene and protein expression analyses. Such hitherto unprecedented effect induces an increased paracellular permeability to FD-4, which appeared to be faster and higher than that observed after LPS and ethanol treatments of the Caco-2 monolayer, along with no observed reversibility. This effect of PA implies a potentially damaging role to gut integrity that needs to be carefully evaluated in order to understand the molecular mechanisms underlying the observed PA activity. For instance, whether or not PA interacts with the fatty acid translocase FAT/CD36, which is an integral membrane protein involved in dietary fatty acid absorption on apical enterocyte membranes [84,85], deserves additional analysis in future studies.

In conclusion, based on the present results it can be hypothesized, for the first time, that palmitic acid, along with its immunomodulatory effect, might play a role in several gastrointestinal disorders in which the intestinal permeability is altered, such as IBD, IBS, and celiac disease. Thus, it would be interesting to verify if there is a correlation between people with impaired intestinal permeability and high consumption of PA-rich food in their daily diet.

### Limits and Future Perspectives

Our study has some limitations. First, although Caco-2 cells represent a widely accepted model of the intestinal barrier utilized in many in vitro works, they are also a heterogeneous cell line that do not always express the same characteristics and the identical behavior of the parental cell line, as extensively reviewed by Sambuy et al. [86]. Therefore, such line heterogeneity and the diverse culture-related conditions might influence the expression of these functional characteristics and might help explain how difficult it can be sometimes to compare the results obtained from different laboratories, in the literature. Second, the experimental protocol of this study used the internal controls (i.e., healthy controls with no exogenous chemical insults) in each treatment to normalize the data and to confirm the observed effects; it could be helpful, in the future, to test a protective agent as a further control in order to support these findings. Moreover, our data regarding the effect of PA, at the physiological postprandial intestinal concentration and with a specific time point (i.e., 24 h), could not exclude a cytotoxic effect or oxidative stress under different conditions; thus, it would be interesting to test various concentrations of PA (ranging from low to high doses) in future works, along with different time-points to corroborate the results of this first study. Since this study was based on an in vitro investigation that used a colon carcinoma-derived cell line, and since it is known that PA is mostly absorbed by the small intestine rather than the large intestine, the addition of in vivo evidence from animal or human studies would be helpful to support the potentially harmful effect of PA on the intestinal barrier integrity observed herein. Thus, measuring the concentration of PA in the small and large intestine, after ingestion of PA-containing food, would help to shed further light on this early effect. Overall, there seems to be a concentration-related mechanism of action (i.e., dose-response) and a time-dependent effect of PA on the gut–barrier integrity and function, as previously observed [82]. Finally, to validate our data on the alteration of intestinal permeability in the Caco-2 monolayers, it would be useful to also perform TEER measurements in future studies, as they better reflect the integrity of the tight junctions.

## Supplementary Materials

The following are available online at https://www.mdpi.com/2076-3921/9/5/417/s1, Figure S1: Immunohistochemical analysis of the TJ and AJ proteins with an unchanged expression.
