# Supplementary figures and images for: Palmitic Acid Affects Intestinal Epithelial Barrier Integrity and Permeability In Vitro
Source: Antioxidants (Basel). 2020 May 13;9(5):417. doi: 10.3390/antiox9050417 (PMC7278681; doi:10.3390/antiox9050417)

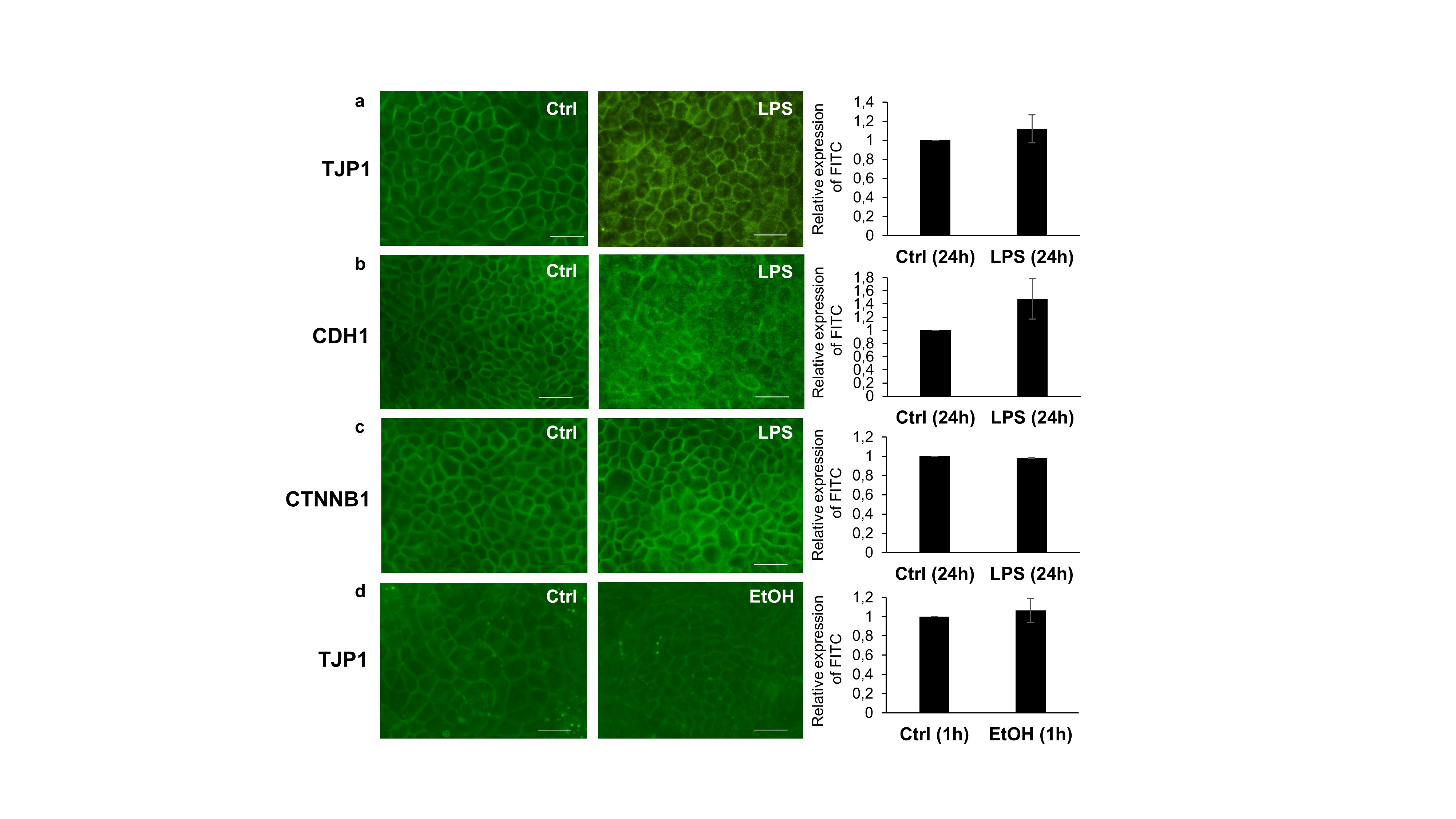

Supplement: Supplementary file 1 [file antioxidants-09-00417-s001.zip › Supplementary Figure 1.jpg]
